# Clinical-stage human anti-transferrin receptor antibodies suppress colorectal cancer metastasis

**DOI:** 10.1038/s41392-026-02917-9

**Published:** 2026-08-20

**Authors:** Mizuki Endo, Takuya Shiraishi, Takehiko Yokobori, Jianyu Lv, Bilguun Erkhem-Ochir, Tadashi Matsuura, Kenichiro Araki, Makoto Sakai, Ken Shirabe, Hiroshi Saeki

**Affiliations:** 1 Department of General Surgical Science, Graduate School of Medicine, Gunma University, Maebashi, Japan; 2 Division of Gene Therapy Science, Gunma University Initiative for Advanced Research (GIAR), Maebashi, Japan; 3 Perseus Proteomics Inc., Chuo-ku, Japan

**Keywords:** Gastrointestinal cancer, Metastasis, Drug development, Tumour biomarkers, Cancer models

**Dear Editor**

Advanced colorectal cancer (CRC) is a leading cause of cancer-related mortality worldwide, with distant metastasis and therapeutic resistance representing critical clinical hurdles.^1^ Transferrin receptor (TFRC), a key iron transporter that is frequently overexpressed in tumors, plays a vital role in tumor progression and is correlated with poor prognosis.^2^ While TFRC inhibition has been shown to suppress growth in various cancers,^3^ its therapeutic effect on the CRC metastatic cascade remains unexplored. While the safety of PPMX-T003 (a clinical-stage, fully human anti-TFRC monoclonal antibody) has been confirmed in phase 1 trials for hematological conditions and a phase 1/2 trial is currently ongoing for aggressive natural killer-cell leukemia,^4,5^ its efficacy against solid tumors remains unknown. To our knowledge, this is the first study to evaluate the efficacy of PPMX-T003 against CRC, providing novel preclinical evidence that direct TFRC inhibition effectively suppresses both primary tumor growth and metastatic spread.

To investigate this, we evaluated the in vivo efficacy of PPMX-T003 using a patient-derived xenograft (PDX) model of CRC liver metastasis. Immunohistochemical analysis demonstrated that TFRC was expressed in both the primary rectal tumor and the corresponding metastasized liver from a patient with CRC whose sample was used to establish the PDX. In addition, histological examination confirmed that the PDX tumors retained the morphological features of colorectal adenocarcinoma and closely recapitulated those of the original patient tumors. These findings support the clinical relevance of our PDX model for evaluating the therapeutic efficacy of PPMX-T003 against CRC liver metastasis. Following subcutaneous implantation, the established tumors were treated with PPMX-T003 or an isotype control IgG1 (Fig. 1a). Two-way repeated-measures ANOVA revealed a significant interaction between treatment and time (F(8, 64) = 2.872; *p* < 0.01; Fig. 1a), indicating that PPMX-T003 can alter the trajectory of tumor growth. The tumor growth curves in the two groups began to diverge from approximately the third measurement onward. Post hoc analysis with Šídák’s multiple comparisons test revealed a significant difference at sacrifice (*p* = 0.0085). Immunohistochemical analysis of excised tumors confirmed that this antitumor effect was associated with a significant reduction in the Ki-67 proliferation index (*p* = 0.0317; Fig. 1a), providing strong evidence for the therapeutic efficacy of PPMX-T003 against established CRC tumors in vivo.

**Fig. 1:**
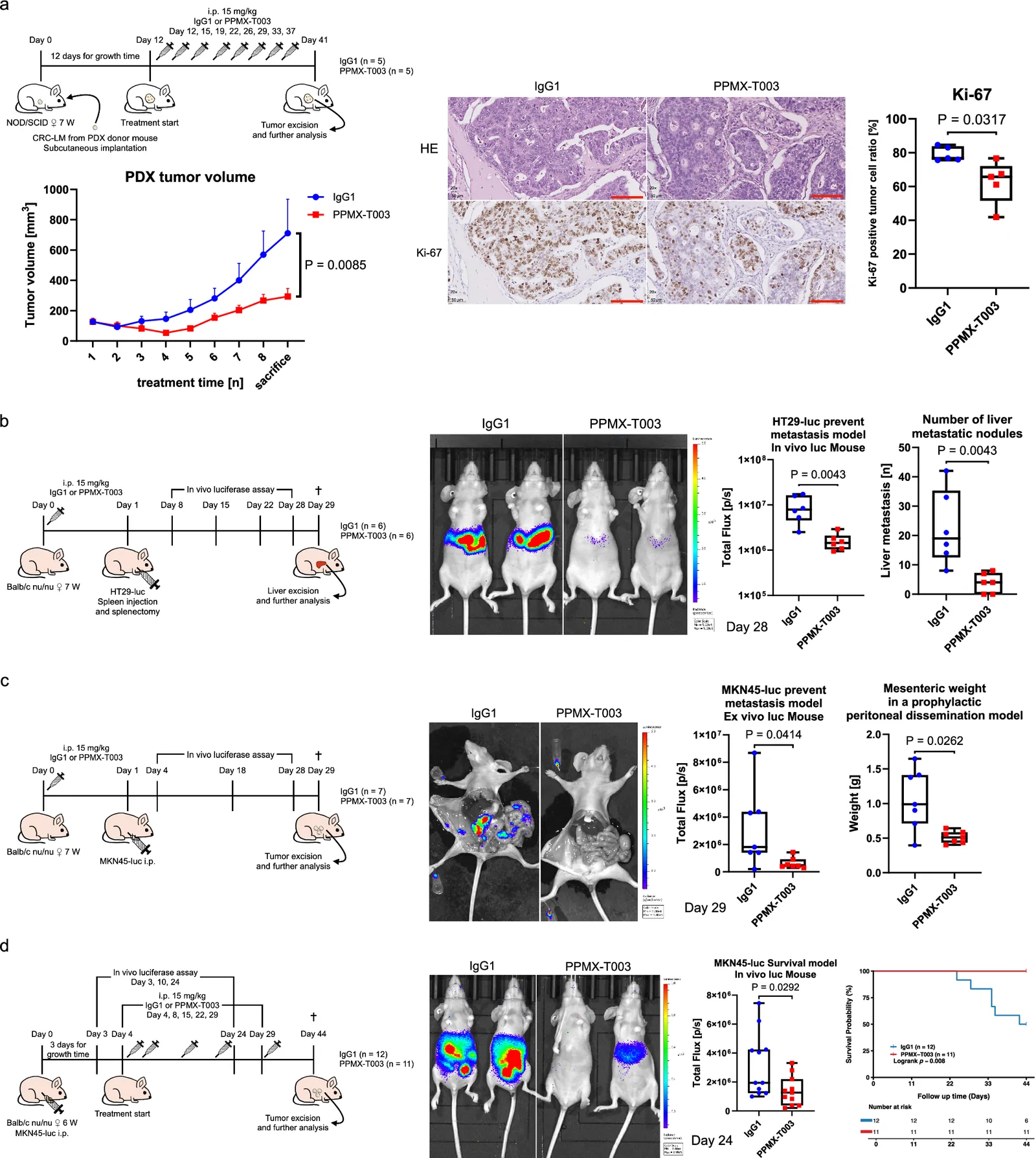
PPMX-T003 inhibits tumor growth and prevents metastatic spread in colorectal liver metastasis and gastric peritoneal dissemination models. **a** Schematic of the in vivo experimental setup. PDX tumors originating from CRC liver metastases were subcutaneously implanted into NOD/SCID mice. Treatment with PPMX-T003 or isotype control IgG1 (15 mg/kg, intraperitoneally, twice weekly) was initiated on day 12. The tumors were harvested on day 41. Tumor growth curves for the control (*N* = 5) and PPMX-T003-treated (*N* = 5) groups. The data are presented as the mean ± SEM. Two-way repeated-measures ANOVA revealed a significant interaction between treatment and time (F(8, 64) = 2.872, *p* < 0.01). Post hoc analysis using Šídák’s multiple comparisons test indicated a significant difference between the groups at sacrifice (*p* = 0.0085 *vs*. control). Representative images of hematoxylin and eosin and immunohistochemical staining for Ki-67 in tumors harvested from both groups (scale bar, 100 μm). Quantification of Ki-67-positive cells is shown. Data are presented as box and whisker plots (where the line in the box indicates the median, the box extends from the 25th to the 75th percentiles, and the whiskers indicate the minimal and maximal values). Statistical significance (*p* = 0.0317) was determined using the Mann–Whitney *U* test. CRC colorectal cancer, LM liver metastasis, PDX patient-derived xenograft, IgG immunoglobulin. **b** Schematic of the splenic injection liver metastasis model. Mice received a single intraperitoneal injection of PPMX-T003 or isotype control IgG1 (15 mg/kg) on day 0, followed by intrasplenic injection of HT29-luc cells on day 1. Their livers were harvested on day 29. In vivo bioluminescence imaging of tumor burden on day 28 and corresponding quantitative analysis of bioluminescence (*p* = 0.0043). The number of liver metastatic nodules significantly decreased in the PPMX-T003-treated group (*p* = 0.0043). For all the quantitative data, *N* = 6 mice/group. Data are presented as box and whisker plots (where the line in the box indicates the median, the box extends from the 25th to the 75th percentiles, and the whiskers indicate the minimal and maximal values). Statistical significance was determined using the Mann–Whitney *U* test. IgG, immunoglobulin. **c** Schematic of the prophylactic peritoneal dissemination model. Mice received a single intraperitoneal injection of PPMX-T003 or isotype control IgG1 (15 mg/kg) on day 0, followed by an intraperitoneal injection of MKN45-luc cells on day 1. The mice were monitored, and their tissues were harvested on day 29. Ex vivo luciferase imaging of intraperitoneal tumor nodules at necropsy on day 29 and corresponding bioluminescence quantification revealed a significant reduction in the number of viable disseminated lesions in the PPMX-T003-treated group (*p* = 0.0414). Mesenteric tissues were harvested at necropsy and weighed. A significant reduction in mesenteric weight was observed in the PPMX-T003-treated group compared with the isotype control IgG1 group (*p* = 0.0262). For all the quantitative data, *N* = 7 mice/group. Data are presented as box and whisker plots (where the line in the box indicates the median, the box extends from the 25th to the 75th percentiles, and the whiskers indicate the minimal and maximal values). Statistical significance was determined using the Mann–Whitney *U* test. IgG, immunoglobulin. **d** Schematic of the therapeutic peritoneal dissemination model. Mice were intraperitoneally injected with MKN45-luc cells on day 0, followed by repeated intraperitoneal administration of PPMX-T003 or an IgG1 isotype control (15 mg/kg) on days 4, 8, 15, 22, and 29. In vivo bioluminescence imaging of tumor burden on day 24 and corresponding quantitative analysis of bioluminescence (*p* = 0.0292). Kaplan–Meier analysis of overall survival in the lethal peritoneal dissemination model. PPMX-T003 treatment significantly prolonged survival compared with that in the control IgG group (*p* = 0.008). For all the quantitative data, *N* = 11 mice/group. Data are presented as box and whisker plots (where the line in the box indicates the median, the box extends from the 25th to the 75th percentiles, and the whiskers indicate the minimal and maximal values). Statistical significance was determined using the Mann–Whitney *U* test. Survival analysis was performed using the log-rank test. IgG immunoglobulin

Having established the efficacy of PPMX-T003 against established tumors, we next addressed the critical challenge of preventing metastasis. We utilized a stringent splenic injection model of liver metastasis in which mice were pretreated with a single dose of PPMX-T003 or control IgG 1 day prior to intrasplenic injection of HT29-luc cells (Fig. 1b). Pretreatment with PPMX-T003 led to considerable inhibition of liver metastasis. In vivo bioluminescence imaging on day 28 revealed significantly reduced tumor signals in the PPMX-T003-treated group (*p* = 0.0043; Fig. 1b). These findings were confirmed by necropsy, where macroscopic examination revealed a marked reduction in the number of visible liver tumor nodules (*p* = 0.0043; Fig. 1b). These findings, together with the marked reduction in tumor number, suggest that the primary mechanism of PPMX-T003 in this prophylactic setting is the potent inhibition of metastatic seeding and colonization rather than the suppression of proliferation in already established micrometastases. Collectively, these results demonstrate the potent antimetastatic effect of PPMX-T003 therapy, which targeted the most lethal aspect of CRC progression.

Having demonstrated the antimetastatic effect of PPMX-T003 in a hematogenous liver metastasis model, we next investigated whether this prophylactic efficacy could be extended to other cancer types and metastatic routes, particularly peritoneal dissemination, a clinically prevalent and lethal mode of metastasis, and whether it could prolong survival in a lethal tumor-bearing model. We therefore established a peritoneal dissemination model using the gastric cancer cell line MKN45-luc. Mice were pretreated with a single intraperitoneal dose of PPMX-T003 or control IgG 1 day prior to intraperitoneal injection of tumor cells (Fig. 1c). On necropsy on day 29, ex vivo luciferase imaging of intraperitoneal tumor nodules revealed a significant reduction in bioluminescence signals in the PPMX-T003-treated group (*p* = 0.0414), accompanied by a significant decrease in excised peritoneal tumor weight (*p* = 0.0262; Fig. 1c).

We evaluated the therapeutic efficacy of PPMX-T003 in a lethal peritoneal dissemination model. A schematic representation of the experimental design is shown in Fig. 1d. Mice were intraperitoneally injected with the gastric cancer cell line MKN45-luc on day 0, followed by repeated intraperitoneal administration of PPMX-T003 or control IgG on days 4, 8, 15, 22, and 29. PPMX-T003 treatment significantly suppressed tumor progression in this therapeutic setting. In vivo bioluminescence imaging on day 24 revealed significantly reduced tumor signals in the PPMX-T003-treated group (*p* = 0.0292; Fig. 1d). Importantly, survival analysis in this lethal peritoneal dissemination model revealed that compared with control IgG treatment, PPMX-T003 treatment significantly prolonged overall survival (*p* = 0.008; Fig. 1d). Collectively, these results demonstrate that PPMX-T003 has robust antimetastatic effects across organs and metastatic routes, preventing metastatic progression and significantly prolonging survival.

In conclusion, our study demonstrated that PPMX-T003 exhibited consistent antitumor effects in in vivo models and significantly suppressed metastatic colonization in a CRC liver metastasis model. Importantly, PPMX-T003 also inhibited peritoneal seeding in a gastric cancer model and significantly prolonged survival in a lethal dissemination model. These findings indicate that TFRC-targeted therapy may be effective across distinct tumor types and metastatic routes, including both hematogenous and transcoelomic metastases. Given that TFRC plays a central role in iron-dependent tumor growth and survival, its inhibition represents a rational strategy for controlling metastatic progression. As a clinical-stage antibody, PPMX-T003 has significant translational potential, and our findings support its further clinical development as a metabolism-based therapeutic approach for metastatic cancer.

## Supplementary information

Supplementary_Materials

## Data Availability

All data supporting the findings of this study are included within the research article.
