# Supplementary material for: Clinical-stage human anti-transferrin receptor antibodies suppress colorectal cancer metastasis
Source: Signal Transduct Target Ther. 2026 Aug 20;11:338. doi: 10.1038/s41392-026-02917-9 (PMC13490405; doi:10.1038/s41392-026-02917-9)
Supplement: Supplementary file 1 [file 41392_2026_2917_MOESM1_ESM.docx]

Supplementary Materials for

Clinical-stage human anti-transferrin receptor antibodies suppress colorectal cancer metastasis

Mizuki Endo, Takuya Shiraishi, Takehiko Yokobori, Jianyu Lv, Bilguun Erkhem-Ochir,

Tadashi Matsuura, Kenichiro Araki, Makoto Sakai, Ken Shirabe, and Hiroshi Saeki

Correspondence to: [bori45@gunma-u.ac.jp](mailto:bori45@gunma-u.ac.jp)

**Summary**

**This PDF file includes:**

Materials and Methods

Materials and Methods

Study design

This study was designed to evaluate the therapeutic efficacy of PPMX-T003 against colorectal cancer (CRC). To this end, we assessed the antitumor and antimetastatic activities of PPMX-T003 using a series of *in vitro* assays, *in vivo* patient-derived xenograft (PDX) and CRC liver metastasis mouse models, and *ex vivo* human CRC liver tissue cultures. In addition, a prophylactic peritoneal dissemination model and a lethal peritoneal dissemination model of gastric cancer were employed to evaluate the antimetastatic effect and survival-prolonging efficacy of PPMX-T003. The primary endpoints were inhibition of tumor growth, suppression of metastatic colonization, and modulation of proliferation markers, such as Ki-67.

Cell lines

Human CRC (HT29-luc) and gastric cancer (MKN45-luc) cell lines were obtained from the American Type Culture Collection (ATCC; Manassas, VA, USA) and Japanese Collection of Research Bioresources (JCRB) Cell Bank (National Institutes of Biomedical Innovation, Health and Nutrition, Osaka, Japan). All cell lines were authenticated via Short Tandem Repeat (STR) profiling at the JCRB Cell Bank. We routinely confirmed that the cultures were *Mycoplasma*-free using MycoStrip (InvivoGen, San Diego, CA, USA). The cells were cultured in Dulbecco's modified Eagle medium (DMEM; Wako, Osaka, Japan) or Roswell Park Memorial Institute 1640 (RPMI-1640; Wako, Osaka, Japan) supplemented with 10% fetal bovine serum and 1% penicillin–streptomycin at 37°C in a humidified 5% CO_2_ atmosphere.

Immunohistochemical staining

Paraffin-embedded CRC specimens, xenograft tumors, and *ex vivo* tissues were sliced into 4-µm–thick sections, which were incubated at 60°C for 60 min and deparaffinized using ClearPlus (FALMA, Tokyo, Japan). Rehydration was performed by immersing the sections in a series of ethanol solutions, followed by antigen retrieval using Immunosaver (Nishin EM, Tokyo, Japan) at a temperature range of 98°C–100°C for 45 min. To inhibit endogenous peroxidase activity, the sections were treated with a solution of 0.3% H_2_O_2_ in 100% methanol for 30 min. Subsequently, the sections were blocked using Protein Block Serum-Free Reagent (Agilent, Santa Clara, CA, USA) and incubated with primary antibodies against REAL Antibody Diluent (Agilent) for 24 h at 4°C. The following primary antibodies were used: anti-Ki-67 rabbit monoclonal antibody (1:200 dilution) (CST-9027; Cell Signaling Technology, Danvers, MA, USA) and anti-TFRC rabbit polyclonal antibody (1:250 dilution) (HPA028598; Sigma-Aldrich, St. Louis, MOS, USA). Visualization of the primary antibody was conducted using a Histofine Simple Stain MAX-PO (Multi) Kit (Nichirei, Tokyo, Japan), following the manufacturer’s instructions. The chromogen 3,3'-diaminobenzidine tetrahydrochloride was applied at a concentration of 0.02% in 50 mM ammonium acetate–citrate buffer (pH 6.0) containing 0.005% H_2_O_2_. Finally, Mayer’s hematoxylin was used for counterstaining. Negative control sections without primary antibody showed no detectable staining.

Establishment of the CRC-PDX model and treatment with PPMX-T003

All animal experiments and procedures were approved by the Animal Care and Experimentation Committee of Gunma University (approval numbers: 23-052 and 23-069) and conducted in accordance with institutional guidelines. A patient-derived xenograft (PDX) model was established with sample from a 66-year-old man with pStage IVa rectal adenocarcinoma and metachronous liver metastasis. Pathological evaluation revealed a moderately differentiated tubular adenocarcinoma (tub2 > tub1) with deep mural invasion (pT4a) and nodal involvement (pN2a; 5/21 nodes positive). Notably, the specimen was collected from a chemo-naive patient during hepatic resection, ensuring that TFRC expression levels represented the baseline metabolic state without treatment-induced clonal selection. Molecular profiling revealed a *KRAS* G12D mutation, while the tumor was negative for *BRAF* V600E, *HER2*, and *MSI* (MSS phenotype). Immunohistochemical analysis confirmed stable TFRC expression across the primary tumor and liver metastasis. For the therapeutic study, fresh viable tumor tissue from donor PDX mice was cut into ~5-mm cubes and subcutaneously implanted into the dorsal region of 7-week-old female NOD/SCID mice (CLEA Japan, Tokyo, Japan) (N = 12; mean body weight ~19.7 g). Tumor formation was assessed 12 days after implantation. Ten mice (83.3%) developed subcutaneous tumors and were included in the therapeutic study. Two mice that did not develop tumors were excluded.

The mice were assigned to either a control group or treatment group (N = 5 mice/group) such that the mean tumor volume was similar between the groups. No significant difference in mean body weight was observed between the treatment and control groups (20.2 *vs*. 19.8 g, respectively). PPMX-T003 or isotype control IgG1 (15 mg/kg body weight) was administered intraperitoneally twice a week. Tumor volume was measured using calipers every 3–4 days and calculated as follows: Tumor volume = (longest diameter × shortest diameter^2^) / 2. Mice were monitored for tumor growth and signs of distress. In accordance with humane endpoint criteria, the mice were euthanized by cervical dislocation under deep isoflurane anesthesia (5%) if the largest tumor diameter exceeded 25 mm; tumors showed signs of ulceration, infection, or necrosis; or mice exhibited persistent signs of distress (e.g., hunching). Surviving mice were euthanized on day 41 post-implantation, and tumors were excised for histological and immunohistochemical analyses. The experimental design and workflow are presented in Fig. 1a.

Evaluation of immunohistochemical staining in xenograft model samples

Immunohistochemical analysis was performed on tumor tissues derived from patient- and cell line-derived xenograft models. TFRC expression was evaluated following the same criteria as described for clinical CRC specimens. For Ki-67 immunoreactivity, five representative tumor regions were selected from each sample. The total number of tumor cells and the number of Ki-67-positive nuclei were automatically quantified using Halo image analysis software (Visualix, Hyogo, Japan). The Ki-67 labeling index was calculated as the percentage of Ki-67-positive nuclei. For the PPMX-T003-treated group, where tumor formation was suppressed, six distinct tumor regions were selected from liver sections where tumor tissue was still pathologically identifiable, and the Ki-67 labeling index was evaluated using the same method. All tissue slides from patient- and cell line-derived xenograft models were digitized using PidPort (Medmain Inc., Fukuoka, Japan) and analyzed on the Halo platform.

Evaluation of the antimetastatic effect of PPMX-T003 pretreatment in a splenic injection CRC liver metastasis model

Seven-week-old female BALB/c-nu/nu mice (CLEA Japan) were used to establish the CRC liver metastasis model. Mice were assigned to two groups (N = 6 mice/group) of equal body weight (mean body weight: 21.38 *vs.* 21.35 g for the PPMX-T003 and isotype control IgG1 groups, respectively). One day prior to tumor cell injection, mice received a single intraperitoneal injection of either PPMX-T003 (15 mg/kg body weight) or isotype control IgG1. The following day, a CRC liver metastasis model was established by intrasplenic injection of 2.0 × 10^6^ HT29-luc cells in 100 µL of phosphate-buffered saline (PBS) under general anesthesia. To prevent tumor formation at the injection site, the spleen was surgically removed 5 min after cell inoculation. Following tumor injection, the mice were monitored for 28 days. All mice survived until the end of the study on day 28; at this point, they were euthanized for the evaluation of liver metastases. The livers were harvested, weighed, and subjected to histological examination and quantitative analysis of metastatic burden. Bioluminescence imaging of live mice and excised liver tissues was performed using IVIS Lumina Series III (PerkinElmer, Waltham, MA, USA). For *in vivo* imaging, AkaLumine-HCl (808350; Sigma-Aldrich) was administered intraperitoneally (5.0 mM; 100 µL/mouse). The experimental design and workflow are shown in Fig. 1b.

Evaluation of the prophylactic effect of PPMX-T003 in a peritoneal dissemination model of gastric cancer

Seven-week-old female BALB/c-nu/nu mice (CLEA Japan) were used to establish the peritoneal dissemination model. The mice were assigned to two groups (n = 7 mice/group) with comparable body weights (20.33 g for the isotype control IgG1 group and 20.31 g for the PPMX-T003 group). One day prior to tumor cell inoculation, the mice received a single intraperitoneal injection of either PPMX-T003 (15 mg/kg body weight) or isotype control IgG1. The following day, peritoneal dissemination was induced by intraperitoneal injection of 2.0 × 10^6^ MKN45-luc cells in 100 µL of PBS. Following tumor cell injection, the mice were monitored over time and subjected to *in vivo* bioluminescence imaging on days 4, 18, and 28. All mice were euthanized on day 29 for further analysis. At necropsy, intraperitoneal tumor nodules were collected and evaluated using *ex vivo* luciferase imaging. Mesenteric tissues were also harvested and weighed as an indicator of tumor burden. *In vivo* bioluminescence imaging was performed using the methods and reagents described for the splenic injection model. For *ex vivo* imaging, 1 mL of AkaLumine-HCl (500 µM in saline) was applied directly onto the surface of the mesentery and peritoneum of mice prior to imaging. The experimental design and workflow are shown in Fig. 1c.

Evaluation of the therapeutic efficacy of PPMX-T003 in a lethal peritoneal dissemination model

Six-week-old female BALB/c-nu/nu mice (CLEA Japan) were used to establish a lethal peritoneal dissemination model. On day 0, peritoneal dissemination was induced by intraperitoneal injection of 2.0 × 10^6^ MKN45-luc cells in 100 µL of PBS. Three days after tumor inoculation, the mice were stratified into two groups based on body weight and baseline bioluminescence quantified via *in vivo* luciferase assay: isotype control IgG1 (n = 12; mean body weight 19.95 g and total flux 1.03 × 10^6^ p/s) and PPMX-T003 (n = 11; mean body weight 19.97 g and total flux 1.10 × 10^6^ p/s).

The mice received repeated intraperitoneal injections of PPMX-T003 (15 mg/kg body weight) or isotype control IgG1 on days 4, 8, 15, 22, and 29. Tumor progression was monitored using *in vivo* bioluminescence imaging at the indicated time points. For endpoint analysis, the mice were euthanized on day 44. *Ex vivo* luciferase imaging of intraperitoneal tumor nodules was performed at necropsy. Mesenteric tissues were also harvested and weighed as an indicator of tumor burden. Bioluminescence imaging procedures were performed as described for the prophylactic model. The experimental design and workflow are shown in Fig. 1d.

Statistical analysis

All statistical analyses were conducted using JMP (version 18.2.2; SAS Institute, Cary, NC, USA) and GraphPad Prism 11 (Dotmatics, Boston, MA, USA). Normality was assessed using the Shapiro–Wilk test. Homogeneity of variances was assessed using Levene’s test. For *in vivo* animal studies, non-parametric tests were employed to account for inherent biological variability and small sample sizes. For these data, two-group comparisons were performed using the Mann–Whitney *U* test, and comparisons among three or more groups were performed using the Kruskal–Wallis test followed by Dunn’s multiple comparisons test. Longitudinal tumor growth data were analyzed using a two-way repeated measures ANOVA with a mixed-effects model, followed by Šídák’s multiple comparisons test. Associations between categorical variables were assessed using Pearson’s Chi-squared test. Data are presented as mean ± standard error of the mean or as box-and-whisker plots. Results with *p* < 0.05 were considered statistically significant.
